# A Comparative Study on the Effectiveness of Positive Psychotherapy and Group Cognitive-Behavioral Therapy for the Patients Suffering From Major Depressive Disorder

**Published:** 2012

**Authors:** Negar Asgharipoor, Aliasghar Asgharnejad Farid, Hamidreza Arshadi, Ali Sahebi

**Affiliations:** 1 Department of Clinical Psychology, Mashhad University of Medical Sciences, Mashhad, Iran.; 2 Department of Clinical Psychology, Tehran University of Medical Sciences, Tehran, Iran.; 3 Azad Islamic University, Mashhad Iran.; 4 William glasser Institute, Sydney, Australia.

**Keywords:** Group Cognitive-Behavioral Therapy, Major Depressive Disorder, Positive Psychotherapy

## Abstract

**Objective**: Aim of this experimental study is evaluating the effectiveness of two different approaches towards the treatment of major depressive disorder (MDD): Positive-oriented psychotherapy and group cognitive-behavior therapy.

**Methods**: Eighteen out-patients suffering from major depression were randomly divided into two groups to be treated according to either of these two approaches. Both groups undertook the treatments for 12 weeks. All the subjects were tested by Beck Depression Inventory, Subjective Wellbeing Scale, Oxford test of Happiness, and the scale of Subjective Units of Distress before and after the treatments.

**Results**: The results show significant differences between the two groups in terms of the variables of happiness and mental distress, suggesting that effectiveness of positive psychotherapy is more than cognitive-behavioral therapy in increasing happiness. These two approaches were significantly different in neither decreasing the acuteness of depression symptoms nor increasing subjective wellbeing.

**Conclusion**: As a whole, the results of this comparative study indicate that positive psychotherapy is more effective in increasing happiness among MDD patients.

## Introduction

According to the data obtained from epidemiologic studies, majordepressive disorder has been reported as the most common and prevalent psychological disorders. So, it is necessary to take the best remedial approach to deal with depressive disorder due to the remarkable economical, social and emotional burden on the part of the sufferers, their families and society from one hand and an estimate of 2.9% to 12.6% of annual prevalence among the general population, on the other hand. According to the recent evidence, depressive disorder, in terms of pathology, is comparable to basic diseases like cancer and arteriosclerosis (1).

The people suffering from major depressive disorder do not have the right attitude towards themselves, the world around them, and the present and future time. Besides, these people possess a low level of life-satisfaction and wellbeing while happy people are more successful in different aspects of life such as marriage, health, job and relationship with others. In many studies, the authors have arrived at the conclusion that positive emotions lead to positive consequences (2).

Recent studies in the field of clinical psychology show interest, not only in studying the effects of negative occurrences and negative characteristics of personality, but also in the fact that the lack of positive attitudes and conditions in life play an important role in depressive disorder (3). The results of a study on 5566 people with low level of wellbeing show that they are 7.16 times more susceptible to be inflicted by depressive disorder over a period of ten years. According to the same study, such people are more likely (as twice as much) to get depressive disorder even if variables like having a history of depression, personality characteristics, negative function, demographic variables and physical health are controlled. This study suggests that the lack of wellbeing and positive emotions can be considered as a risk factor for depressive disorder (4).

The existence of high level of wellbeing along with positive emotions is of crucial importance in people’s life satisfaction. Research studies show that those who experience and express positive emotions are more likely to conduct a happy life with more rewarding interpersonal relations and achieve their goals in life. In addition, such people are physically healthier and expect a longer life (5).

Positive psychology was pioneered by Martin Seligman. Positive psychology encourages psychologists to develop an open-minded outlook towards people’s strengths, aptitudes and capabilities. In other words, it scientifically studies the optimum functions of human beings. The subject of positive psychology concerns with three domains as associated to three aspects of life. The first domain is called “pleasant life” which deals with positive emotions about the past, present and future. Positive emotions about the past life consist of satisfaction, intimacy and peace of mind. The positive emotions about future consist of hopefulness, optimism and trust. As a whole, “pleasant life” is the life which increases positive emotions while decreasing the negative ones. The second domain refers to as “engaged life” which draws on the positive individual characteristics like strengths and virtues. Strengths are those qualities which have always been considered as values by different cultures and over historical eras, such as affection, common sense, the capacity of loving and being loved. The third domain of positive psychology is called “meaningful life” which is concerned with the feeling of belonging and giving service to positive institutes and societies believing that positive emotions and characteristics can best develop in such settings, leading to meaningful life. As a whole, these three types of life are regarded as three paths leading to happiness (6).

Fredrickson et al, (2000) point out the implications of positive psychology in various dimensions such as correction, improvement and optimizing methods of psychotherapy; correction and improvement of family life as well as correction, improvement and optimizing the positive features in the different constitutes of a society. Thus, positive psychology is a recognized movement towards directing humans into development, flourishing and pride. Also, Seligman views positive psychology as an efficient method of therapy believing that certain interventions should be designed to create happiness and positive functions, assuming that positive functions are different from negative functions (7).

Although there are some research findings emphasizing the effectiveness of cognitive-behavioral therapy in the treatment of major depressive disorder, the number of studies attempting to compare this kind of therapy with positive interventions is scare. There is only one case study which was carried out in one month and proved that positive psychotherapy had better results in reducing the level of depressive disorder, as compared to treatment as usual (8).

The present research is important in that it is one of the rare studies which aim at investigating the effectiveness of positive psychotherapy in the treatment of major depressive disorder through a comparison with cognitive-behavior therapy. Another importance of this study is making use of group therapy which is advantageous over individual significant therapy for obliging everybody in the group to change, having a stronger form of observational learning, meeting people with identical problems, and having access to a range of individuals to be used against cognitive errors.

## Materials and Methods

This study adopted purpose-oriented sampling for selecting accessible samples. The psychiatrists were provided with the criteria of inclusion and exclusion, based on which the qualified patients were selected and referred to a therapist to be considered for the preliminary evaluation. Having conducted SCID tests on the patients, the therapist first had the selected patients complete the conscious consent agreement. Then, those qualified patients who formally gave consent to participate in the research were selected as the sample of the study to be evaluated while receiving the treatments. The treatments were consulted and coordinated with the psychiatrists so that the subjects of two groups would undertake identical pharmaceutical therapy with the same prescribed dose of medicine. (All of patients of two groups were received citalopram with average dosage of 20-40mg).One month after initiation of pharmaceutical therapy,psychotherapy sessions of two groups were initiated.As all patients received similar dosage of citalopram,therapeutic response is result of employed psychotherapy method.The subjects were randomly divided into two experimental groups.The two groups were not significantly different regarding demographic variables and severity of depressive symptoms.Also, two groups were homogeneous according to time of onset of major depressive disorder. One of the experimental groups was exposed to positive psychotherapy while the other experimental group received group cognitive-behavior therapy. The subjects attended two-hour sessions per week for a total of twelve weeks meeting in Pardis Centre of Consulting and Psychological Services in Mashhad. Two groups were performed by a unique therapist. Before and after the treatments, the subjects took Beck depression inventory, subjective wellbeing scale, Oxford test of happiness, and subjective units of distress scale. With using MANCOVA for analyzing the data, the possible effects of pretest scores of variables (severity of depression, score on SUDS, happiness and subjective wellbeing test) and demographic variables controlled. Also, there were no significant differences between groups regarding demographic variables at baseline. Therefore, both groups are alike with regard to different variant.

*Inclusion criteria*

The inclusion criteria adopted for this study are as follows:

- The patients must have major depressive disorder(mild, moderate or severe) criteria as identified by DSM IV – TR in axis I
- The patients must hold a degree of junior high school, at least
- The patients must be in the age range of 20 to 40 years old
- The patients must not have any history of psychotherapy

*Exclusion criteria*

The patients were excluded from the study due to the following criteria:

- Being identified with psychotic symptoms
- Being addicted to allsubstances
- Having the complete criteria as defined for other disorders in axis I
- Being mentally engaged in serious thoughts of committing suicide
- Having the complete criteria as defined for personality disorders in axisII

*Structured Clinical Interview for DSM IV*

Structural Clinical Interview is a diagnostic instrument devised based on Diagnostic Statistical Manual of mental disorders, fourth edition (DSM IV). In order to conduct this interview, the interviewer needs to make judgments around the interviewee’s responses. Therefore, the interviewer must have the knowledge and experience of psychopathology (9). This instrument is available in two versions. The first version is SCID-I which deals with major mental disorders (axis I in DSM IV). This study used the translated and adapted form of this instrument by Sharifi et al. (2005)(10).The other version used by this study is SCID-II which was translated and adapted by Bakhtiari (2000)(11). The second version deals with the evaluation of personality disorders(i.e. axis II in DSM IV).

*Questionnaire of demographic characteristics*

In order to collect data about the demographic characteristics of the population of the study, the researcher devised a questionnaire to find out about their age, sex, marital status, job, education, place of residency and the past history of MDD.

*Beck Depression Inventory-second edition (BDI-II)*

As a self-reporting instrument, BDI-II is used for assessing the factors involved in depressive disorders. This inventory includes 21 statements regarding various symptoms of depression (12). The revised version of this inventory (BDI-II), as compared to its first version, is more adaptable to DSM IV covering all the elements of depressive disorders based on cognitive theory. Like the first edition, the second one consists of 21 items with four response choices which indicate the level of depression. The items are scaled from zero to 3 which make an overall range of 0-63. There is no cut off point predicted by the inventory for no depression. The cut off points suggested for this inventory are: scores of 0-13 indicating minor depression; 14-19 suggesting mild depression; 20-28 showing moderate depression; and the score range of 29 to 63 which demonstrate severe depression (13). Having been translated and conducted on 125 Iranian university students, BDI-II has been reported to have Alfa coefficient of 0.78 and retest coefficient of 0.73, with a two-week interval. This study used the copy translated by Ghasemzadeh et al.

*Subjective Units of Distress scale (SUDS)*

SUDS is a self-assessment scale used for ranking the degree of distress units. It has been scaled in a range from 0 to 10. A score of 0 indicates lack of distress whereas a score of 10 represents the highest level of distress (14). Using this scale, the therapist requires the patient to rank their degree of distress based on a numerical scale. This scale is one of the commonest scales which has been experienced by individuals in the domain of cognitive-behavior therapy and represent improvement during therapy(15). This study used this scale to determine the degree of distress among the participants. 

*Oxford test of happiness*

This scale questionnaire was produced by Argyle, Martin and Gressland in 1989 and was revised in 2001. It includes 29 four-choice questions. The researchers who have analyzed the component items of this scale in different experiments confirm the validity of its structure. This scale includes 7 factors of happiness as: positive cognitive process, social engagement, positive emotion, feeling of control, physical balance, self-satisfaction and mental awareness. Several studies have reported the reliability and validity value of the questionnaire as desirable. For instance, the evaluative studies carried out in England, United States, Australia and Canada have respectively reported the reliability coefficients of 0.89, 0.90, 0.89 and 0.89 for this questionnaire (16). In Iran, too, according to Alipour& Harris (2007), Oxford test of happiness has very good internal consistency, with a Cronbach alpha coefficient reported of 0.91. Also, in order to determine the reliability value of the test, they performed factor analysis, using principal component. The factor load of the items, in extraction factor, was calculated as 0.39 to 0.79. (17).

*Subjective Wellbeing Scale*

This questionnaire evaluates the dimensions of wellbeing in terms of emotional, psychological and social domains. So, there are three subscales as follows:

*Emotional wellbeing subscale:*

It consists of 12 questions which evaluates the positive and negative emotions on the part of the respondents during the month before taking test. This self-performance questionnaire requires the participants to report the duration of positive and negative emotions that they have experienced during the last 30 days. Based on a Likert scale, the respondents rank their emotions from one (never) to 5 (always). In other words, one means the worst emotional situation while 5 represents the best feeling they have experienced. 

*Psychological wellbeing subscale:*

This subscale includes 18 questions in 6 components such as: self-acceptance, personal development, purpose in life, dominance over the environment, autonomy, and positive relationship with others. The respondents evaluate themselves based on Likert scales ranging from 1 (Totally disagree) to 7 (Totally agree).

*Social wellbeing subscale:*

There are 15 questions in 5 components: social acceptance, social realism, social participation, social association, social unity and integration. The respondents rank their general self-evaluation about social relations on a Likert scale ranging from 1(Totally disagree) to 7 (Totally agree).

The reliability and validity of subjective wellbeing scale has been tested on various samples by Keyes & Magyarmu (2003). The internal consistency of emotional wellbeing subscale was calculated as 0.91for positive emotions and 0.87for negative emotions. The subscales of psychological and social wellbeing were estimated to have the average internal consistency of 0.4 to 0.7 with a total of 0.8 for both subscales.

Golestanibakht (2007) has translated this questionnaire and investigated its psychometric features on a group of Iranian subjects. According to this study, the reliability of subjective wellbeing scale was estimated 0.7 and the emotional, psychological, and social subscales obtained reliability values of 0.76, 0.64 and 0.76, respectively. Also, Cronbach alpha coefficient was reported 0.8 for subjective wellbeing scale and those of 0.86, 0.80, 0.64 for emotional, psychological and social subscales, respectively(18).

This study used the positive psychotherapy protocol compiled by Dr. Ali Sahebi(2011)(19). The first session was an orientation session in which the participants were acquainted with the rationale of the treatment as well as the responsibilities of the therapist and the clients. In the second session, the participants practiced how to identify their potential capabilities and strengths and were assigned to look for ways of strengthening those potentials afterwards. The third and fourth sessions focused on the ways of appreciating positive affairs in life. In the fifth session, the participants were introduced to four life styles: Nihilism, pleasure-seeking, competition and happiness (four coordinate axes) and their role in achieving happiness. The participants were then required to write down their experiences under each of these axis. In the sixth, seventh and eighth sessions, they were instructed how to produce their life map and how to rank their daily activities based on pleasure and meaningfulness as well as noting down the time allotted to each activity. The clients were then asked to work on the hierarchy of values in a way that each one of them was required to make a list of his/her highest values. Finally, they were required to coordinate life styles with the highest rank of values in the hierarchy.

As for group cognitive-behavioral treatment, the first six sessions were specified to using the behavioral techniques such as, welcoming and orientation, self-awareness, self-caring, daily activity scheduling, and behavioral assignments. During the last six sessions, the participants were acquainted with the cognitive techniques including teaching ABC technique, describing negative automatic thoughts and cognitive distortions, downward arrow, testing beliefs, logical analysis, self-reward and self punishment.

## Results

Nine of the subjects were assigned to the positive psychotherapy treatment and the other nine subjects comprised cognitive-behavioral group. The mean and standard variation (SD) of the participants’ age were calculated as 26.44 and 6.10, respectively. There were 2 males and 7 females in the positive psychotherapy group while the cognitive-behavioral group consisted of 3 males and 6 females. In both groups, 5 of the participants were single and the rest were married )Table 1).

**Table 1:** Demographic variables of two groups

	**Positive psychotherapy**	***CBT***	**p.value**
**Age**	26 (±3.64)	26.88(±8.10)	0.36
**Sex**			0.50
Male	02(%22. 2)	03(%33.3)	
Female	07(%7.77)	06(%66.7)	
**Education**			0.26
Diploma	02(%22.2)	05(%55.6)	
Master	06(%66.7)	04%)44.4)	
MA and higher	01(%11.1)	00	
**Marital status**			0.68
Single	05(%55.6)	05(%55.6)	
Married	04%)44.4)	04%)44.4)	

**Table 2:** Mean and SD of depression, happiness, wellbeing and SUDS scores of pre and post-treatment stage of two groups (Positive Psychotherapy and Group Cognitive-Behavioral Therapy

**Stages** **Groups**	**Measures**	**Pre test**		**Post test**	
		**Mean**	**SD**	**Mean**	**SD**
**Depression**	Positive	032.55	09.73	017.11	10.81
	CBT	024.33	08.39	011.44	06.71
**Happiness**	Positive	021.11	05.98	042.11	06.39
	CBT	023.55	07.10	030.55	06.02
**Wellbeing**	Positive	164.66	16.89	176.44	10.89
	CBT	156.88	20.01	181.22	18.45
**SUDS**	Positive	008	01	004.77	01.56
	CBT	008.6	01	002.66	01.58
**Emotional wellbeing**	Positive	033	04.66	032.77	02.68
	CBT	032.77	04.6	036.55	03.74
**Psychological wellbeing**	Positive	007.55	07.24	083.88	12.78
	CBT	074	10.64	082.55	11.20
**Social wellbeing**	Positive	054	11.57	059.77	04.89
	CBT	050.11	12.52	062.11	09.41

**Table 3:** Multivariate tests of significant differences among groups on a combination of dependent variables

**Effect**	**Value**	**F**	**Sig** **.**	**Observed power**
**Group**				
Pillai’s Trace	00.915	8.98	0.015	0.89
Wilks’ Lambda	00.085	8.98	0.015	0.89
Hotelling’s Trace	10.77	8.98	0.015	0.89
Roys largest root	10.77	8.98	0.015	0.89

**Table 4:** Between-subjects variance analysis regarding dependent variables

**Source**	**Dependent variable**	**Mean square**	**df**	**F**	**Sig** **.**	**ES**	**Observed power**
**Group**	BDI	025.73	1	0.38	0.54	0.03	0.087
	wellbeing	003.08	1	0.015	0.90	0.001	0.051
	suds	018.75	1	7.43	0.021	0.42	0.69
	happiness	295.90	1	7.2	0.023	0.42	0.68
	emowellbeing	001.30	1	0.31	0.59	0.03	0.08
	Psyc wellbeing	059.62	1	0.47	0.50	0.04	0.095
	Soci wellbeing	023.28	1	0.35	0.56	0.03	0.084

As for data analysis, Multivariate Analysis of Covariance (MANCOVA) was used to have a control over the possible effects of the scores of the variable obtained before the treatments (severity of depression, amount of SUDS, scores on happiness and wellbeing test) on those obtained after the treatment. MANCOVA is used for comparing groups on a range of different characteristics, especially when we have more than one dependent variable. One of the pre-assumptions of using MANCOVA is homogeneity of variances which was met for this study by running Levene’s Test. Then, the data were analyzed.

The mean scores and standard variations at the post-treatment stage are tabulated in table 2. Table 3shows the results of multivariate tests. A set of multivariate tests of significance like Pillai’s Trace, Wilks’ Lambda, Hotelling’s Trace and Roys largest rootindicate that there are statistically significant differences among the groups on a combination of dependent variables. In other words, the vectors of mean scores are significantly different between the groups of study. The results of the test of between-subjects variance analysis are shown in table 4, indicating that the two groups are significantly different in terms of the variables of happiness and subjective distress. Regarding the findings in table 4, there is significant difference between two groups regarding increasing happiness and according table 2 (mean score of happiness at post-treatment stage in positive group is larger than CBT group) it can be concluded that positive psychotherapy group outnumbered cognitive-behavioral group in respect to increasing happiness. As for the variable of subjective units of distress, there was significant difference between the two groups of the study, too. However, the study groups did not show any significant differences in terms of general subjective wellbeing and its subscales of emotional, psychological and social. In addition, there was no significant difference between the two groups in relation to decreasing the rate of depression.

## Conclusion

The findings of this study show a significant difference in the effectiveness of two psychotherapy approaches; i.e. positive psychology and cognitive-behavioral treatments in increasing the level of happiness among the sufferers from major depressive disorder. A review of literature suggests no research study ever done on comparing the effectiveness of these two approaches on major depressive disorder. However, in line with the results of the present study, there are some studies (20,21) which have similarly come up with the positive effects of positive psychotherapy on increasing happiness in a way that the intervention group were found happier than the control group who did not receive any treatment.

By the same token, the findings of the present study are congruent with those obtained from previous studies suggesting positive effect of cognitive-behavioral treatment on the improvement ofsubjective distress. For instance, Ball, et al (2000) compared the effectiveness of group cognitive- behavioral method and assertiveness groups on depression. The results of their study suggest the decrease of subjective distress and depression in both groups(22). Similarly, Davidson, et al (2004) showed in their study that cognitive-behavioral therapy reduces the level of mental disorders among the patients suffering from social phobia(23).Nevertheless, since such studies did not compare cognitive-behavioral with positive psychotherapy regarding the possible effects on subjective distress, the present study remains unique.

According to the findings of this study, there was no significant difference between the two methods of therapy regarding the effectiveness on the reduction of depression symptoms. There are a good number of studies which have pointed out the positive effect and the advantage of cognitive-behavioral therapy over the other methods, dealing with depressed patients(22,24,25). Also, there are several studies suggesting that positive psychotherapy, either performed individually or in group, has significantly positive effect on reducing depression and increasing subjective wellbeing among the depressed patients (26,8).However, there is no study carried out to compare these two approaches in respect to reducing depression symptoms.

The reason why this study found no significant difference between the two approaches, that is positive psychotherapy and group cognitive-behavioral treatments, can be attributed to the fact that the skills of increasing pleasure, engagement and meaningfulness, as the components of positive psychology, can function as complements to the skills of cognitive-behavioral therapy. Thus, it can be concluded that these two approaches have something in common in the treatment of depression. In addition, while comparing two active treatments with each other, the degree of specificity might decrease (6).

Furthermore, the findings of this study indicate no significant difference between positive psychotherapy and cognitive-behavioral approaches in increasing the level of general subjective wellbeing and/or its components; i.e. emotional, psychological and social wellbeing. According to a meta-analytical study concerning 51interventions of positive psychology on 4266 participants, such interventions significantly lead to the increase of subjective wellbeing (mean r = 0.29) as well as the decrease of depression symptoms (mean r = 0.31). However, due to the scarcity of published quantitative studies in the discipline about the general effectiveness of positive psychology and the comparison with other methods of treatment, one cannot make a frank judgment about the efficiency of this approach, yet. On the other hand, studies show that variables such as individual characteristics and the instruments used may affect the efficiency of positive psychology intervention. For example, an increase of age range, individual’s having a choice of participating in groups and an increase of the length of the duration of intervention are among the factors leading to better effects on the participants’ raise of subjective wellbeing (27).So, this matter can be considered as a limitation for this study. Besides, there are other limitations concerning the subjective wellbeing scale, used in this study, which may account for the lack of significant difference between the two groups. For example, some of the items in the questionnaire may be either ambiguous or redundant.

As a whole, it can be concluded that there is a significant difference between positive psychotherapy and cognitive-behavioral approaches in increasing happiness and decrease of subjective units. However, no significant difference was observed in terms of other variables of the study, such as reducing the acuteness of depression and the increase of subjective wellbeing. It seems that using positive activities, as complements to cognitive-behavioral skills, may more effectively help the patients suffering from major depressive disorder to decrease their level of depression and optimize their wellbeing. It goes without saying that more accurate investigation requires further longitudinal studies of longer duration of time.

And last, but not least, the researcher admits that the small size of sample and lack of follow-ups are among the limitations of this study. Thus, it is recommended that the further studies investigate the matter with larger sample size and follow up the results more carefully.

## Authors’ contributions

NA conceived and designed the evaluation, collected clinical data, performed parts of statistical analysis and drafted the manuscript. AAF participated in interpreting clinical data and revised the manuscript and performed parts of analysis. HA participated in collecting and interpreting data. AS re-analyzed the clinical and statistical data and revised the manuscript. All authors read and approved the final manuscript.

## Notes

**Registration ID in IRCT** : IRCT 201201268829N1
